# Environment and health impacts of synthetic food packaging: materials, synthesis, causes, and potential solutions

**DOI:** 10.1039/d6ra02694j

**Published:** 2026-08-03

**Authors:** Md. Amir Khasru, Tarikul Islam, Sayam, Md Shakirul Islam

**Affiliations:** a Department of Yarn Engineering, Barishal Textile Engineering College Barishal 8200 Bangladesh; b Department of Textile Engineering, Jashore University of Science and Technology Jashore 7408 Bangladesh; c Department of Textiles, Merchandising, and Interiors, University of Georgia Athens GA 30602 USA tarikul@uga.edu; d Department of Fabric Engineering, Barishal Textile Engineering College Barishal 8200 Bangladesh; e Department of Textile Engineering, Chemistry, and Science, North Carolina State University Raleigh NC 27696 USA

## Abstract

Food-packaging materials protect food from spoilage by enhancing its shelf life, safety, and overall quality. Currently, a major portion of food-packaging materials are made from fossil-based synthetic polymers due to the cost and ease of processing; however, they have far-reaching consequences on the environment and human health. Their multifaceted impact on the environment is not only limited to carbon footprint throughout their life cycle but also worsened by microplastic release affecting the aquatic systems. The non-biodegradability of such petroleum-based polymers is exacerbating the waste generation upon disposal, negatively affecting soil fertility and raising massive landfill issues. The processing materials and functional additives that can migrate into food and pose toxicological risks also raise concerns. This state-of-the-art review briefly outlines the traditional production processes of available commercial food-packaging materials from different polymeric materials and additives prior to explaining their consequences and broader impacts on the environment and human health in detail. It further highlights promising alternatives, emphasizing biodegradable and renewable polymers paired with safer bio-based additives to maintain the functional performance. The paper concludes with a forward-looking perspective on advancing sustainable and environmentally responsible packaging solutions across industrial sectors.

## Abstract 2

Synthetic food packaging harms the environment and human health through plastic pollution, microplastics, and chemical migration—where biodegradable polymers and bio-based additives offer sustainable alternatives for future food packaging.

## Graphical abstract

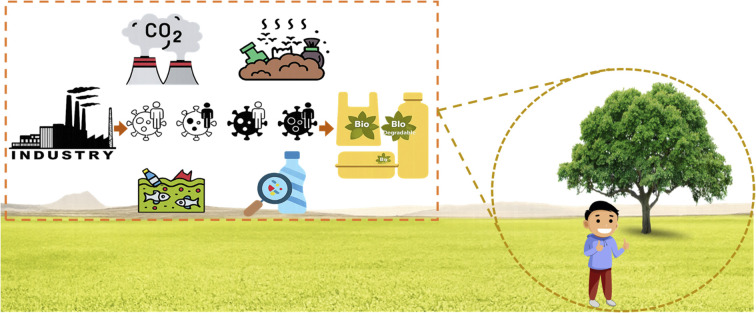

## 1 Introduction

Since ancient times, packaging materials have been essential to carry, exchange, and preserve goods. In 1500 B.C., jars and bottles made of ceramic and glass were designed for food and beverages to control humidity and oxygen in Egypt.^1^ In China, treated mulberry bark was used to wrap foods for centuries before the birth of Christ. Later in the early 19th century, tin cans and heat-sealed paperboards were commercially used for food packaging.^2^ With the invention of cellophane and aluminum foil, flexible food packaging became very popular.^3^ Today, this evolution culminates in lucrative, functional, even sometimes involving multi-material, multi-layer packaging systems engineered to protect food, extend shelf life, and support distribution.

Industrial food-packaging materials have three layers, and these are the primary packages, secondary packages, and tertiary packages. Primary packages cover food from the inner surfaces; secondary packages compact them together for market supplies, and tertiary packages prevent foods from outer damages.^4^ For example, to prevent outside damage and influence, foods are covered with different packaging materials, like glass, metals, aluminum foils, tinplate, steel, paper, paperboards and plastics.^5^ However, the modern food-packaging industry is mostly dominated by plastics derived from petroleum-based sources that are non-biodegradable. Potential applications of non-biodegradable synthetic materials in food packaging have created environmental pollution concerns.^6^

Plastic waste from food packaging is one of the sources of landfill issues, and this accumulated waste needs hundreds of years to decompose.^5^ Additionally, the synthesis of these plastics and the packaging production process emit significant amounts of carbon dioxide and other greenhouse gases that might contribute to rising temperatures and global warming.^7^ In 2022, the EU produced 16.16 million tons of packaging where only 40.7% were recycled.^8^ In 2023, around 50% of solid waste in the USA was due to food-packaging waste and more than 40% of global plastics were used for packaging. Moreover, these plastics are often combined with plasticizers, fillers, pigments, and other additives to make packaging materials, which are harmful upon release into the environmental system. From the European Environment Agency Report of 2022, approximately 14 million tons of microplastics have been released into the world's ocean floor. The release of microplastics from packaging materials is now another pressing issue. Microplastics are solid plastic pieces—fragments, films, pellets, or fibers—smaller than 5 mm.^9^ In practice, many technical sources bracket the “micro” size class at roughly 1 µm to 5 mm. In 2016, annually 8 million tons of plastics were released to the ocean environment.^10^ Microplastic released from such packaging contains hazardous chemicals, 50% of which are still unknown, and the degradation of microplastics is still undefined. As a result, microplastics gradually accumulate in minerals, crops, water, milk, seafood, and other components of the food chain, posing serious risks to human and animal health.^7^

Microplastics cause many health effects with risk of death and have been associated with many health issues such as lung cancer, cardiovascular and respiratory diseases, DNA damage, and cellular damage.^7^ Microplastics influence the immune system and could weaken the defense mechanisms of the body. They affect human hormones, cause hormonal imbalance, reduce sperm quality, and also have an impact on ovarian function in females.^11^ According to some research data of 2015, 7 out of 7 species of turtles, 81 out of 123 species of mammals, and 203 out of 406 species of seabirds were affected by microplastic pollution, containing 29.1% of polyethylene, 18% of polypropylene, 20% of polyethylene terephthalate and 1% of polyamide.^7^ These growing issues can be mitigated by the development of sustainable food-packaging materials from degradable biobased plastics.^12^

**Fig. 1:**
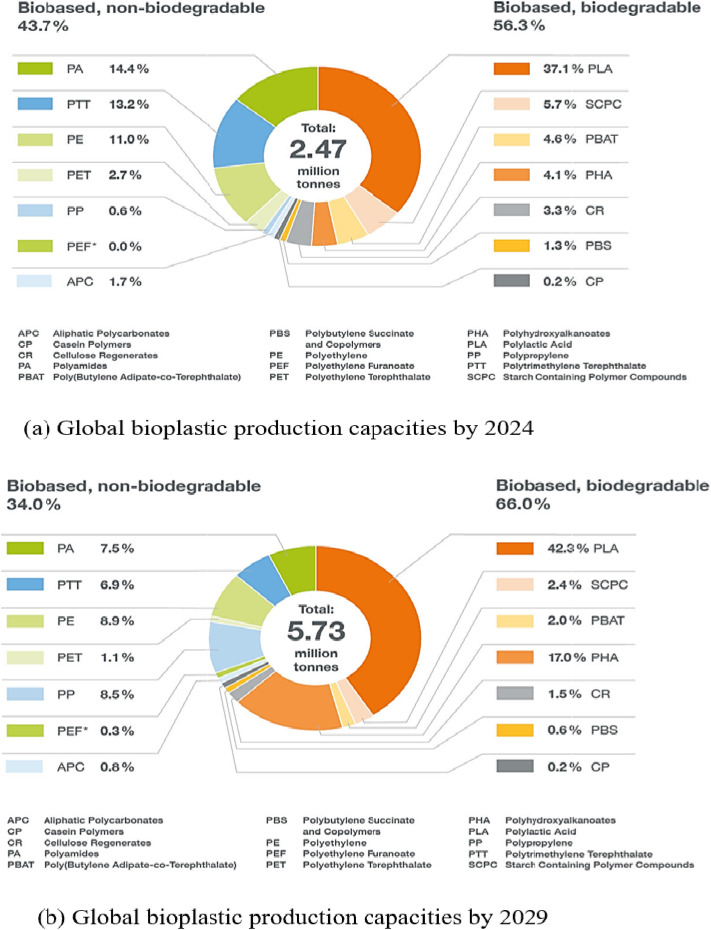
Global bioplastic production capacities (a) forecasted by 2024 and (b) forecasted for the year 2029. Published under the CC-BY License.^13^ Copyright 2024, the authors. Published by the European Bioplastics.

Bioplastics are plastics that are derived partly or wholly from biomass and/or designed to biodegrade under defined conditions and are pivotal to realize the sustainable food-packaging industry. The global production of bioplastics was 2.47 million tons in 2024, and the production forecasted for 2029 is 5.73 million tones, as shown in Fig. 1. Interestingly, not all bioplastics are degradable under certain conditions, *i.e.* 50% of the total bioplastics are not biodegradable. Biodeterioration, depolymerization, bio-assimilation, and mineralization are the four steps of biodegradation of the process. Mineralization converts fragmented materials into carbon dioxide, water, and biomass. Again, the conditions of biodegradation of different bioplastics are not the same.^12^ Several parameters, such as concentration of enzymes, microorganisms, temperature, pH value, humidity, oxygen supply, and light, dictate the degradation process. Therefore, designing packaging materials from bioplastics or bio-derived plastics requires careful considerations.^12^

This review article highlights the potential impact of synthetic food-packaging materials made from non-degradable conventional polymers on the environment and health. To mitigate this, several polymeric packaging materials produced from biological raw materials were used, and their typical production process is first briefly discussed. Then, as a part of solution, biodegradable, industrially viable, biobased polymers with environment-friendly additives for potential packaging applications and their synthesis process are explained. While other studies focus on the adverse consequences of the petroleum-based plastics derived from packaging, this study illustrates relevant constituent materials and the mechanisms associated with such impacts before suggesting safer materials to mitigate negative consequences. Additionally, this study also discusses the technical features of alternatives, helping the industry to design scalable, sustainable packaging materials.

## 2 Packaging materials

### 2.1 Types of packaging materials

**Fig. 2:**
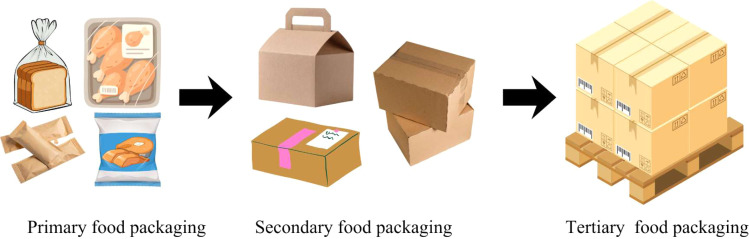
Schematic of the primary, secondary, and tertiary food packaging (created with Canva).

Packaging materials are of three types: primary, secondary and tertiary packaging, as shown in Fig. 2. Primary packaging is directly in contact with the products, defending the first line of environmental factors, including vials, ampoules, blister packs, and bottles. Secondary packaging contains primary packaged products, provides additional protection, display branding and information, and includes cartons and boxes, which can hold more units of primary packages. Tertiary packaging is used for bulk handling of products, storage, and shipping, including wooden pallets, larger containers, such as drums, and barrels that contain multiple units of secondary packaging.^14^ Polyethylene (PE), polypropylene (PP), polyamide (PA), PET (polyethylene terephthalate), PS (polystyrene), polyvinyl alcohol (PVOH), polyvinylidene chloride (PVDC), polyvinyl acetate (PVAc), poly(ethylene-*co*-vinyl acetate) (PEVA), polycarbonate (PC), polyvinyl chloride (PVC), and poly(ethylene-*co*-acrylic acid) (PEAA) are commonly used polymers in food packaging.^15^ PE and PP, are more widely used for solid food packaging and PET for liquid food packaging. Liquid food-packaging materials have higher impacts on the environment than others, beverage industry also impacts higher.^16^ Biodegradable packaging materials can be made from edible films, coatings, and other bio food-packaging sources.^17^

### 2.2 Raw materials for food packaging

Traditional food packaging provides protection and physical support to a food product from external environments. Food packaging also protects from heat, light, moisture, pressure, oxygen, enzymes, microorganisms, odors, insects, dust, and dirt.^18^ Metal glasses, paper, and polymers are being used in food packaging to save food from the damages.^19^ Synthetic raw materials such as PE, PP, PET, poly(trimethylene terephthalate) (PTT), and PA are most widely used in food packaging.^20^ Most polymers are not suitable for use in initial stages, and so industries use additives and modifiers to adjust the properties of polymers. Thermal stability, color, odor, surface gloss, and other properties are controlled by additives such as anti-degradants (to inhibit degradation), fillers (for improved mechanical properties), curing agents (which help to form a three-dimensional network structure) and coupling agents (to enhance the compatibility of components).^21^

#### 2.2.1 Polymeric raw materials

PE is the most widely used food-packaging polymeric material. PE films are heat sealable, strong, tough films with good moisture prevention properties.^22^ Low-density polyethylene (LDPE) is mostly preferred for food packaging.^23^ This thermoplastic polymer is produced by free radical or addition polymerization of ethylene (or ethene) monomers and used in production and consumption. The types of polyethylene include high-density polyethylene (HDPE), low-density polyethylene (LDPE), linear low-density polyethylene (LLDPE), ultra-low-density polyethylene (ULDPE), cross-linked PE, and PE copolymers.^24^ These polymers are found in pellet and powder form and required to melt before processing into a desired packaging shape. Fossil-based PE generates higher greenhouse gas emissions than bio-based PE during production; however, bio-based PE may generate more microplastics than fossil-based PE, which could adversely affect human health. The applications of thin films are in food packaging, plastic containers, bottles, bags, plastic toys, wire and cable insulations, and medical tubing, among others.^25^

PP is a stiff, robust, crystalline thermoplastic polymer, synthesized from the propene monomer by a chain-growth polymerization process. It was first discovered in 1951 by Paul Hogan and Robert Banks, scientists of Phillips Petroleum Company, and first commercialized in 1954.^26^ It is lower in density, cost, high melting point, and good heat sealability.^27^ Low prices with outstanding chemical and physical qualities of PP have drawn attention to the food-packaging industry. PP films with active agents like additives and stabilizers improve the functional properties of polymer films.^28^ Bio-PP is synthesized from bio-based alcohol, including biopropanol dehydrated into bio-propylene. Then, the polymerization of bio-ethylene produces bio-PP.^26^

PET is the third most widely used polymer in the food-packaging industry, primarily derived from fossil sources. It is mainly used in beverage bottles and has become famous for its lightweight and remarkable strength.^29^ It is a thermoplastic polymer, produced from the polycondensation of ethylene glycol and terephthalic acid. Ethylene glycol is derived from ethylene by means of the catalytic oxidation of ethylene with oxygen and terephthalic acid is derived from the oxidation of paraxylene (isolated from the catalytic reforming of petroleum as BTX aromatics fraction). Feedstocks of ethylene glycol are ethanol, glycerol, sorbitol, biomass and terephthalic acid are ethanol, hydroxymethylfurfural (HMF), isobutanol, biomass, isoprene/acrylic acid, limonene, furfural, PET bottles and plastics, which can be easily recycled after being used and discarded. Almost 87% of bottled water sold in 2016 is preserved in PET containers, and therefore, PET is called the king of the bottles for drinks. Generally, PET is not biodegradable, but it can be degraded *via* enzymatic reactions involving bacteria/enzymes properly isolated.^30^ Biomass-based PET have 21% less global warming potential and require 22% less fossil fuel than their fossil-based PET. The applications of PET are in bottles, packaging materials, textile fibers, medical tubing, textiles, electronic components, construction materials, automotive components, and diagnostic tools.^31,32^

Poly(trimethylene terephthalate) (PTT), poly(trimethylene isophthalate) (PTI), poly(trimethylene naphthalate) (PTN), and their copolymers are aromatic polyesters prepared by the polycondensation of 1,3-propanediol (PDO) with terephthalic acid (TPA), isophthalic acid (IPA), or naphthalenedicarboxylic acid (NDA), with additional comonomers (*e.g.*, isophthalic acid or 1,4-butanediol) incorporated to modify their properties.^33^ PTT is a thermoplastic with high mechanical strength, toughness, and fatigue resistance which is widely used in bottles.^34^ Carbon fiber-reinforced materials show characteristics such as light-weight, high strength, corrosion resistance, high thermal stability, and easy material processability. Recently, scientists have been focusing on developing low-cost biobased composites using recycled carbon fibers and PTT. It could be used in apparel, including jeans, trousers, shirts, white athletic socks, carpet, and upholstery.^35^

PA is used in food packaging and various surface treatments such as UV, plasma, and corona applied to extend the shelf-life of food.^36^ It is mainly used in food-sensitive multilayer packaging films to protect from the reaction of oxygen. PA is a polymer which contains an amide group (CO–NH) in a molecular chain. PA can be made by combining a diamine with a dicarboxylic acid or by self-condensation of amino acid or amino acid derivative. Bio-based PA is derived from proteins and peptides which are naturally occurring polymers that are made up of amino acids. PA are divided into three categories: aliphatic, semi-aromatic, and aromatic polyamides. Polyamides are also known as aramids.^37^ Polyamides have good mechanical, electrical, and thermal properties and show high electrical and temperature resistance. Types of polyamides include Nylon 6, Nylon 6,6, Nylon 6,10, Nylon 11, and Nylon 12, which are applied in the automotive sector and textile fiber.^38,39^

Table 1 summarizes some physical, mechanical, thermal, electrical, environmental, and end-of-life properties of common synthetic polymers used in food-packaging applications. PE and PP are characterized by comparatively low densities (approximately 900–960 kg m^−3^), which explains their widespread use in lightweight packaging, whereas PET and rigid polyvinyl chloride (PVC) exhibit significantly higher densities above 1350 kg m^−3^, reflecting their more compact polymer structures.^40,41^ Mechanical stiffness varies substantially among the materials: PE shows the lowest Young's modulus, indicating high flexibility, while PET and rigid PVC demonstrate markedly higher stiffness, making them suitable for rigid containers and structural packaging components.^41,42^ Tensile strength follows a similar trend, increasing from PE to PP and reaching higher values for PET and polyamides, whereas elongation at break highlights the ductile nature of PE and PP in contrast to the brittle behavior of PS, which exhibits minimal elongation before failure.^41,42^

**Table 1:** Polymer properties of synthetic food-packaging materials

Category	Property	Polyethylene (PE, mainly HDPE/LDPE)	Polypropylene (PP)	Polyethylene terephthalate (PET)	Polyamides (nylons, PA 6/6,6)	Polyvinyl chloride (PVC, rigid)	Polystyrene (PS, GPPS/HIPS)	Ref.
General	Density (kg m^−3^)	910–960	900–910	1350–1410	1120–1150	1350–1450	1040–1060	42 and 43
Mechanical	Young's modulus (GPa)	0.2–1.0 (grade-dependent)	1.0–1.6	3.5–11	1.5–3.0	2.4–4.1	3.0–3.6	43 and 44
Mechanical	Tensile strength at yield (MPa)	20–35 (HDPE/LDPE films)	30–50	50–75 (bottle/film grades)	60–80	45–60 (rigid PVC)	46–60	43 and 44
Mechanical	Elongation at break (%)	200–900 (LDPE/HDPE)	200–700	2.5–165 (film *vs.* molded)	20–300 (grade-dependent)	20–80 (rigid PVC)	3–7	43 and 44
Thermal	Melting point or softening (°C)	110–135 (LDPE/HDPE)	160–170	250–260	215–265	75–105 (amorphous, *T*_g_/softening)	100 (*T*_g_, amorphous)	43 and 44
Thermal	Typical max service temperature (°C)	80–100	100–110	120–150	120–160	60–80	70–90	43 and 45
Electrical	Volume resistivity (Ω cm)	10^15^–10^19^	10^16^	10^15^–10^17^	10^13^–10^15^	10^12^–10^15^	10^16^	43 and 44
Electrical	Dielectric constant (1 kHz)	2.2–2.3	2.2–2.3	3.0–3.4	3–4	3–4	2.5–3.0	43 and 44
Electrical	Dissipation factor (1 kHz)	≤10^−3^	2 × 10^−3^–5 × 10^−3^	1.6 × 10^−2^	0.01–0.03	0.01–0.03	0.001–0.003	43, 44 and 46
Eco (material)	Embodied energy (MJ kg^−1^)	70–85	70–80	80–90	80–90	60–80	90–100	46
Eco (material)	CO_2_ footprint (kg CO_2_ per kg)	1.8–2.5	1.8–2.7	2.5–3.5	3.0–4.0	1.9–2.5	3.0–3.8	46
End of life	Heat of combustion (MJ kg^−1^)	43–46	43–46	22–24	25–35	16–20	38–42	46
End of life	CO_2_ from complete combustion (kg CO_2_ per kg)	3.1–3.2	3.1–3.2	2.3–2.5	2.5–3.0	1.9–2.3	3.2–3.5	46
Recycling	Typical recycle code	HDPE (2), LDPE (4)	PP (5)	PET (1)	PA (7)	PVC (3)	PS (6)	42–44

Thermal properties further differentiate these polymers in packaging performance. PE and PP soften or melt at relatively low temperatures, while PET and polyamides maintain structural integrity at substantially higher temperatures, enabling their use in hot-fill and thermally demanding applications.^41,42^ PVC and PS, despite moderate stiffness, show lower thermal resistance, limiting their service temperature range.^41,43^ From an electrical standpoint, all polymers display high volume resistivity, typical of insulating materials, although PET and polyamides exhibit higher dielectric constants and dissipation factors than PE and PP, indicating increased dielectric losses.^41,42,44^ Environmental indicators reveal comparable embodied energy values across the polymers, with PS and PET generally positioned at the higher end, while CO_2_ footprints are lowest for PE and PP and highest for polyamides, reflecting differences in raw material processing and polymer synthesis routes.^44^ End-of-life characteristics show that PE and PP possess higher heats of combustion than PET and PVC, which is relevant to energy recovery, whereas PVC generates lower combustion energy and reduced CO_2_ emissions per unit mass.^44^ Recycling classifications further distinguish these materials, with established resin codes for PE, PP, PET, PVC, and PS, while polyamides are typically grouped under mixed or other plastic categories, complicating large-scale recycling streams.^40–42^

#### 2.2.2 Additives

Additives are chemical substances contained in all plastic products for enhancing the polymer properties. Additives may be released from plastics during the recycling and recovery process.^45^ Additives are used in polymers to improve the physical and chemical properties of packaging polymers. Additives are normally used in polymer materials to gain required properties according to users, but some additives contain heavy metals (lead, cobalt, nickel, copper, *etc.*).^46^ Food additives are added to food during processing, manufacturing, or storage to enhance appearance, texture, and preservation, and also play a critical role in food safety with standards.^47^ However, some scopes for the avoidance of additives remain, like controlling food degradation process, microbiological spoilage, chemical degradation, physiological damage, gas balance, and ethylene control.^48^ Traditional food packaging focuses on the shape and size of the package in which food safety is ensured by adding synthetic preservatives. Active food packaging also focused on food safety, temperature maintenance, oxidization, microbial attract reduction, *etc.*, which ensure powerful food protection.^49^ Plant materials such as lignocellulosic fibers, nanocellulose, or lignin are used in plastic materials as additives.^50,51^ Additives allow thermal processing to reduce polymer degradation and maintain properties. In the earlier stage, oils, fats, and hydrocarbons were used in packaging to avoid oxidation during storage. Bisphenol-A, butylated hydroxytoluene, and other phenolic antioxidants are identified as antioxidant additives.^52^ Plasticizer, an additive, is added to a material to make the material softer or more flexible, and it also used to reduce the melt viscosity, the glass transition temperature, and/or the elastic modulus and lower the temperature of a second-order transition.^21^ Commonly plasticized materials are cellulose acetate and cellulose nitrate.^53^ To enhance the texture, consistency, and shelf life of food products, stabilizers are applied in food packaging. Hydrocolloids, proteins, emulsifiers, and antioxidants are the most common types of additives which are used in ice cream, sauces, beverages, and dressings, to improve their sensory attributes and to prevent separation.^54^ Microbial stabilizers have been designed to improve the food shelf life in recent studies.^55^

### 2.3 Manufacturing techniques of packaging materials

The manufacturing techniques of food-packaging materials can be largely divided into two methods: melting and solvent casting. Synthetic semi-crystalline polymers are melted, whereas polymers that do not have melting points or have melting points closer to degradation temperature usually dissolved in suitable solvents for solvent casting. The melt-casting process includes extrusion, injection molding, blow molding, rotational molding, and thermoforming.^56^

**Fig. 3:**
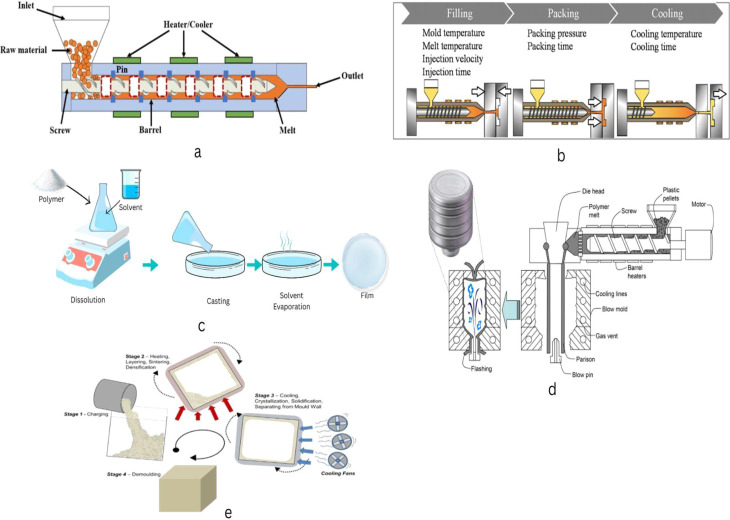
Schematic of the (a) extrusion process (published under the CC-BY License.^58^ Copyright 2024, the authors. Published by MDPI), (b) injection molding (reproduced with permission from ref. 59 Copyright 2022, Springer Nature), (c) solvent-cast polymeric film where the polymer is first dissolved into a solution, followed by casting the solution into a suitable medium and then drying it in a controlled environment (created with Canva), (d) blow molding (reproduced with permission from ref. 60. Copyright 2017, Elsevier), and (e) rotational molding (published under the CC-BY License.^61^ Copyright 2024, the authors. Published by MDPI).

In the extrusion process, as shown in Fig. 3a, plastics are transformed from solid state to liquid state and solidified again without losing the main properties. Raw polymers in the form of powder, pellets, and eventually milled scrap are brought to the fluid state by heating and forcing it to pass continuously through a shaped profile, called extrusion head or die, where it takes the form, and then cooling it to give the desired shaped and stabilize the mold. As demonstrated in Fig. 3b, injection molding, a cyclical and discontinuous process, shapes thermoplastic materials by melting and injecting them into a mold to form the desired shape This method is widely used to produce household goods, bottles, and mechanical components.^57^

Solvent casting is more suitable for polymeric materials that degrade before melting or does not have melting temperature, as illustrated in Fig. 3c. In this process, polymers are first dissolved into compatible solvents, often accompanied by additives to enhance process or modify properties.^62^ A thin layer of solution is then spread on a flat surface or mold and allowed to dry under controlled conditions.^63^ As a result of this process, uniform, transparent films with controlled thickness are produced, which are widely used in food-packaging applications.^62^ While some synthetic polymers, or blends can be made using solvent casting methods, mostly biodegradable or renewable polymers are manufactured by this process.^64^ In recent times, more research has been conducted with a focus to utilize bio-derived biodegradable polymers in food-packaging applications.

Blow molding combines extrusion and blow molding technologies and is generally used for manufacturing flasks and bottles, as shown in Fig. 3d. It is a plastic forming process where a molten plastic is first passed through an open mold and then it closes and cuts the tube. Compressed air helps to expand and take the shape of the mold, and after cooling, hollow plastic items such as bottles are formed. Fig. 3e presents the rotational molding process of hollow plastic product production. Thermoforming is a fabrication process, where raw plastic sheets are heated to the softening point and shaped through vacuum or pressure. Using vacuum or pressure, thermoforming plastics can be shaped into different complex shapes. The application of thermoforming plastics are in automotive, packaging, electronics, and consumer goods.^57^

## 3 Impacts on environment

Food-packaging materials are developed from either petroleum sources or bio-based sources including corn, food waste, wood, and vegetables. PE, PP, PS, PC, PVC, and PET are derived from petroleum sources, PLA from corn, sugarcane, cassava, and wheat (starch).^65^ PHA from food waste, vegetable oils, and wood sugars, PBS from petroleum or corn/sugarcane, PBAT from petroleum, and polysaccharide from corn, potato, cassava, and wheat.^66^ The manufacturing stages of food-packaging materials require an energy supply from the raw material to the final product, as illustrated in Table 2, which ultimately accounts for 30% of global energy consumption.^67^

**Table 2:** Energy consumption and production data for plastics

Polymer	Total energy demand (MJ kg^−1^)	Process energy (MJ kg^−1^)	Feedstock energy (MJ kg^−1^)	Ref.
PE-LD	64.6–92	8.53 (polymerization)	43–55	68 and 69
PE-HD	70–94	5.43 (polymerization)	55	68 and 69
PP	64–111.5	—	44–58	68 and 69
PVC	52.4–79.5	—	18–24	68 and 69
PS	70.8–118	—	40	68 and 69
PET	71.2 (virgin); 109–115 (processed)	38–44 (processing)	24	68 and 69
PC	78.2–117.4			68 and 69

**Fig. 4:**
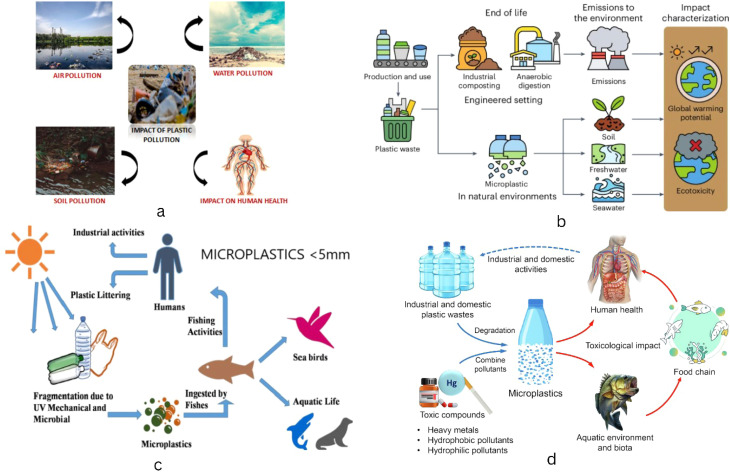
Schematic of (a) impacts of plastic pollution on air, water, soil, and human health (published under the CC-BY License.^70^ Copyright 2021, the authors. Published by JPAM), (b) microplastic and toxic particle emission flow, with the impact of plastic particle release on the environment (published under the CC-BY License.^71^ Copyright 2024, the authors. Published by Springer Nature), (c) plastic life cycle from plastic to micro-plastic release (reproduced with permission from ref. 72 Copyright 2021, Springer Nature), and (d) impacts of microplastics on human health (published under the CC-BY License.^73^ Copyright 2025, the authors. Published by Elsevier).

Apart from energy consumption, food packaging has a significant impact on environmental pollution, including but not limited to air, water, and soil pollution.^70^ Large plastic litter from industrial activities enters the environment. Litter undergoes fragmentation due to UV, microbial action, and breaking down into microplastics (Fig. 4a and b).^71^ The discharged material emits pollutants that have significant impacts such as global warming, and fresh water small microplastics are ingested by fishes, affecting seabirds and aquatic life, as illustrated in Fig. 4c.^72^ Pollution occurs in the form of greenhouse gas emissions contributing to global warming, microplastic release, and landfill accumulation, respectively. These toxic particles entered into the food chain, the aquatic environment and biota, and human health, as shown in Fig. 4d. These pollution further cause an impact on human health, leading to complicated diseases and fatal consequences.^73^ This section will highlight the environmental and health impacts caused by food-packaging materials.

### 3.1 Environmental footprint of food packaging

The term “environmental footprint” here refers to the overall environmental impact associated with the plastic food packaging from its manufacturing to end life.^74^ Carbon footprint, water footprint, and microplastics (plastic particles sized from 1 to 5000 µm^3^) have the worst impact on the environment, which led to air, water, and land pollution, respectively, indicated by carbon emission, microplastic release in water, and landfill issues.^75^

In 2015, the plastic was responsible for 4.5% of global greenhouse gas (GHG) emission, and according to growth rates, it will almost quadruple by 2050. In 2020, the plastic sector was responsible for 2.2 gigatons (Gt) of CO_2_, which accounts for 7% of total global energy emissions and is estimated to reach 2 °C of warming target by 2100.^76^ Packaging materials from petroleum-based polymers cause significant carbon emissions accounting for higher carbon footprints. For example, cradle-to-grave carbon emission for typical fossil-based polymers ranges from 1.91 to 2.51 kg CO_2_-eq. per kg polymer, which includes only the production process, let alone its transportation and disposal, as shown in Table 3.^77^ For instance, a life cycle assessment by Choi *et al.* analyzed LDPE packaging films through production, use, and three disposal routes—incineration, landfill, and recycling. The study found that total greenhouse gas emissions ranged from roughly 4 to 7.5 kg CO_2_-equivalent per kilogram of film.^78^

**Table 3:** Greenhouse gas emission factors for polymer types, plastic polymer production, and upstream processes (kg CO_2_-eq. per kg polymer)

Polymer	Emissions due to crude oil production (kg CO_2_ per kg crude oil)	Refinery (CO_2_-eq. per kg crude oil)	Greenhouse gas emissions (kg CO_2_ eq. per kg polymer)	Ref.
PP	0.228	0.34	1.91	79 and 80
PE-LD	0.228	0.34	1.98	79 and 80
PE-HD	0.228	0.34	1.93	79 and 80
PVC	0.228	0.34	2.51	77, 79 and 80
PUR	0.228	0.34	5.70	79 and 80
PET	0.228	0.34	2.94	77, 79 and 80
PS/EPS	0.228	0.34	3.68	79 and 80
Other	0.228	0.34	2.51	79 and 80

The plastic raw material, manufacturing, secondary packaging, transport, and end-of-life stages each contributed 45%, 38%, 5%, 3%, and 9% of the total life cycle greenhouse gases, respectively.^81^ Plastic does not only contribute to carbon footprint but also pollutes terrestrial and aquatic environments such as soil, lakes, rivers, and oceans by intensifying landfill issues, releasing toxic chemicals, and causing health problems. Less than 0.3 metric tons of plastic are estimated on the ocean surface to be circulating and 9 million to 23 million metric tons of plastic emitted annually.^75^ In 2019, more than 380 million metric tons (MMT) of plastic wastes were generated and India produced one-fifth of the total amount. From 1970 to 2019, over 100 MMT of plastic wastes were collected from rivers and seas, as presented in Table 4.^82^ Plastic packaging materials persist for decades in landfills, fragmenting into micro- and nano-plastics that infiltrate soil and groundwater.^83^ Globally, around 79% of all plastic waste had accumulated in landfills in 2015,^84^ where food packaging alone accounts for 40%.^85^

Microplastic footprint in eastern India

**Table:** 

Microplastics found in eastern India			
Sample	pH (minimum range–mean–maximum range)	Microplastic abundance (lowest-average-highest)	Ref.
Pond water	5–5.4–6	59–63–67 particles per L	82
River water	6–6.9–7	88–93–100 particles per L	77
Pond sediment	6–7–7.8	176–182–188 particles per g	77 and 82
River sediment	5.8–6–6.8	167–180–193 particles per g	82

**Table:** 

Microplastic footprint in the urban ponds and rivers of eastern India			
Category	Type	Microplastics (%)	Ref.
Size distribution	Type II (300–1180 µm)	56.98	82
Size distribution	Type I (1180–5000 µm)	43.02	77
Polymer type	Nylon	36	82
Polymer type	PE	25	77 and 82
Polymer type	PET	18	82
Polymer type	PVC	9	82
Polymer type	Polyurethane (PU or PUR)	5	86
Polymer type	PP	5	82
Polymer type	PS	2	86
Shape distribution	Film	39.07	77 and 86
Shape distribution	Fragment	22.2	82 and 86
Shape distribution	Particle	12.63	82 and 86
Shape distribution	Fiber	9.73	77
Shape distribution	Pellet	9.3	82
Shape distribution	Foam	1.7	82

Water footprint is measured by the volume of freshwater used in the whole production process. The water footprint values for PP/GF, PP/KF, PP/JF, PP/CF, PP/PLA, and PP are, respectively, 0.8 m^3^/FU, 2.5 m^3^/FU, 6.0 m^3^/FU, 7.5 m^3^/FU, 9.5 m^3^/FU, and 1.0 m^3^/FU (Table 5). Food packaging releases toxic chemicals and particles such as microplastics, phthalate esters, bisphenols, heavy metals, poly- and per-fluoroalkyl substances (PFAS), fragmentation products, chemical leachates, biofilms, and decomposition products. A report from eastern India shows that the microplastic contents in pond water, river water, pond sediment, and river sediment are, respectively, 59–63–67 particles per L, 88–93–100 particles per L, 176–182–188 particles per g, and 167–180–193 particles per g.^87^

**Table 5:** Water footprint for different composition variants in functional unit (FU)

Composition	Description	Water footprint (m^3^/FU)	Ref.
PP/GF	Polypropylene + glass fiber	0.8	86 and 88
PP/KF	Polypropylene + Kenaf fiber	2.5	86
PP/JF	Polypropylene + jute fiber	6.0	86 and 88
PP/CF	Polypropylene + cotton fiber	7.5	86
PP/PLA	Polypropylene + polylactic acid	9.5	86
PP	Pure polypropylene	1.0	86 and 88

### 3.2 Impact on health

**Fig. 5:**
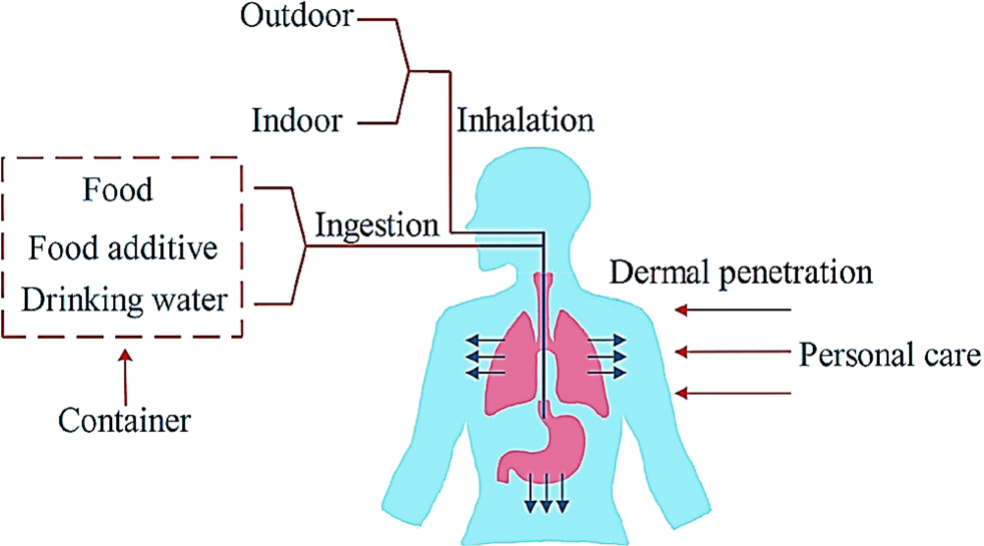
Schematic of the microplastic exposure pathways (created with Canva).

Microplastics and nanoplastics released from food packaging caused health issues such as reproductive organs, placenta and brain,^89^ Food-packaging particles and additives also caused significant health impacts. It has entered the human body in many ways, as shown in Fig. 5, for example, dermal penetration or ingestion. Food additives are ingested through drinking beverages, water, and juice and eating packaged food. Poisonous heavy elements entered into food-packaging materials in various phases of manufacturing. Metals present are cadmium in leak-proof bags, plastic bags, arsenic in paper wrapping and paper board, mercury in paper food packaging, and heavy metals in adulterants and coloring agents used in food packaging.^90^ Low-molecular-weight substances such as plasticizers and antioxidants from food packaging are associated with health issues such as cancer and reproduction problems. Bisphenol A, phthalates, styrene, caprolactam, vinyl chloride, dioxin, paraben, perfluoroalkyl substances, heavy metals, benzophenone, nitrosamine, naphthylamine, benzidine, and 4-aminobiphenyl are toxic migrants found in food packaging, which have significant health impacts.^91^

Table 6 highlights potential diseases caused by microplastics and nanoplastics (MNPs), as reported in several studies. These particles penetrate skin or get into the body through ingestion affecting different organs of the body, leading to genotoxicity, carcinogenicity, respiratory system damage, gastrointestinal tract damage, and genotoxicity. For example, bisphenol and phthalate disrupt the endocrine system, which is essential to regulate the internal functions through hormones.^92^ The disruption of these hormones can cause reproductive and metabolic system dysfunction. Microplastics can even interrupt chromosomes causing blastogenesis, a form of genotoxicity.^93^ The presence of microplastics in human biological matrices, including blood, placenta, and tumor tissues, underscores direct human exposure and systemic health risks. Microplastics and nanoplastics have been found in various human cancer cells including lung, colorectal, gastric, cervical, breast, pancreatic, prostate, and penile malignancies. Zhao *et al.*^94^ detected three types of microplastics: PS, PVC, and PE in lung, gastric, colorectal, and cervical tumors. Xu *et al.*^95^ detected microplastics in human cervical cancer tissue using Raman spectroscopy. Human biomonitoring studies also detected microplastics in nearly 80% of tested human blood samples^96^ and identified 40 microplastic particles across 31 out of 50 placental samples,^97^ confirming direct human exposure and systemic distribution. In cervical cancer tissues, 101 microplastic particles consisting of 12 different polymer types were identified, where PE (26.73%) and polypropylene (19.80%) were the dominant polymers, and exposure levels significantly increased with cancer progression (*p* < 0.05).^98^*In vitro* studies on THP-1 monocyte-like cell lines further demonstrated reactive oxygen species (ROS) generation, oxidative stress, DNA damage, and chromosomal abnormalities following exposure to aged PS microplastics.^99^ Similarly, *in vivo* zebrafish embryo studies revealed neurotoxicity, apoptosis, altered neurotransmitter regulation, and disrupted gene expression after exposure to 500 nm PS microplastics.^100^ Although much of the mechanistic evidence currently originates from controlled laboratory studies, while human evidence remains largely observational and biomonitoring-based, these findings collectively provide strong evidence regarding the potential long-term health risks associated with chronic microplastic exposure.

**Table 6:** Health impacts of microplastics and nano-plastics

Affected system	Potential diseases/conditions	Notes	Ref.
Skin	Contact dermatitis and skin cancer	*In vitro* mechanisms and microplastics promote cancer cell growth through ROS-mtDNA pathways while inhibiting growth in normal skin cells (HaCaT)	101 and 102
Gastrointestinal tract	Inflammatory bowel disease, diabetes, obesity, allergic reactions, and cancer	Positive correlation between fecal microplastics and inflammatory bowel disease (IBD) severity in humans; animal models demonstrate intestinal crypt damage	103
Respiratory system	Chronic obstructive pulmonary disease (COPD), asthma, pneumoconiosis, fibrosis, and alter immune cell profiles	The presence of MNPs is confirmed in human sputum and lung tissue, and COPD-like injury is established in mouse inhalation models	104
Cardiovascular diseases	Cardiovascular diseases, including increased blood pressure, vascular inflammation, and myocardial damage	Epidemiological surveys have also revealed that people exposed to microplastics are more likely to suffer from cardiovascular diseases, such as hypertension and myocardial infarction	105
Neurological effects	Cerebral ischemia, Alzheimer's, Parkinson's, and amyotrophic lateral sclerosis (ALS)*etc.*	A higher plastic burden was found in post-mortem dementia brains, and protein misfolding was observed *in vitro*	106
Reproductive system	Oxidative stress, inflammation, and endocrine disruption affect both male and female reproductive systems	Research on animal models has shown that microplastics impair reproductive cell function, decrease sperm quality, disrupt ovarian function, and reduce fertility	107

#### 3.2.1 Toxicological effects of additives

During manufacturing of food-packaging materials, various chemicals are added to achieve the required properties such as flexibility and heat/flame resistance. Stabilizers, antioxidants, nucleating agents, pigment agents, antistatic agents, and plasticizers are added to food-packaging materials for modification during manufacturing. More than 10 000 chemical additives are linked with polymers, which are detected in marine environments, human food chains, and others. Risks of additives to human health are very alarming and continuously releasing in the environment, which raises significant concerns of biotoxicity including in human beings. These additives are used in 25 different applications, which are typically 0.001% to 50% of the weight (w/w) of the final product in concentration. Chemical additives from food packaging can migrate into food during storage or heating.^91^ Low-molecular-weight additives diffuse from the packaging material into food, especially when affected by temperature, contact time, food composition, and packaging properties. After migration, these compounds may be consumed with food and raise potential safety concerns. For example, endocrine-disrupting chemicals (EDGs) are linked with health effects such as infertility, obesity, diabetes, breast or prostate cancer, thyroid disorders, increased risk of cardiac problems, growth and cognitive impairment, and neurological disorders, as summarized in Table 7. Seref and Cufaoglu (2025) listed different migrant additives with their respective toxicity concentrations;^91^ however, the hazardous effects of >50% of additive chemicals are still unknown.^101,108^

**Table 7:** Toxicological effects of additives

Additives	Chemical compounds	Effect on human health	Ref.
Plasticizers	1,2-Benzenedicarboxylic acid, chlorinated paraffins, dicyclohexyl phthalate (DCHP), di-C7-11-branched and linear alkyl esters (DHNUP), butyl benzyl phthalate (BBP), diethyl phthalate (DEP), dibutyl adipate (DHA), formaldehyde, 4,4′-methylenedianiline (MDA), dipentyl phthalate (DPP), diisobutyl phthalate (DiBP), diisoheptylphthalate (DIHP), and heavy metals (zinc, cadmium, tin, lead, titanium, and barium)	Neuronal toxicity, breast cancer, cardiovascular and kidney diseases, metabolic and mental disorders, and neuro-degenerative disorder	101
Biocides	Arsenic trioxide, triclosan, triphenyltin hydroxide, butyltin trichloride, dimethyltin dichloride, dibutyltin dichloride, tetrabutyltin, tributyltin chloride, and heavy metals (antimony, copper, mercury, arsenic, and tin)	Metal–estrogen, mutagen, carcinogen, brain damage, congenital disabilities, lung, skin, liver, bladder, kidneys, and gastrointestinal damage	101 and 108
Stabilizers, antioxidants and organic pigments	Bisphenol A, fatty acid amides, 2-*tert*-butyl-4-methoxyphenol, triglycidyl isocyanurate (TGIC), 2-*t*-butyl-4 hydroxyanisole (BHA), tris(2,4-di-*tert*-butylphenyl) phosphate, butylated hydroxytoluene (BHT), tris-nonyl-phenyl phosphate (TNPP), 4-nonylphenol, irganox 1010, 4-octylphenol, and heavy metals (aluminum, manganese, barium, cobalt, chromium, lead, titanium, tin, cadmium, and aluminum)	Metabolism changes, DNA methylation, anemia, neurological disorder, cardiovascular and endocrine deficits, hypertension, miscarriages, disruption of nervousness, brain damage, and infertility	108 and 109

## 4 Potential solutions

**Fig. 6:**
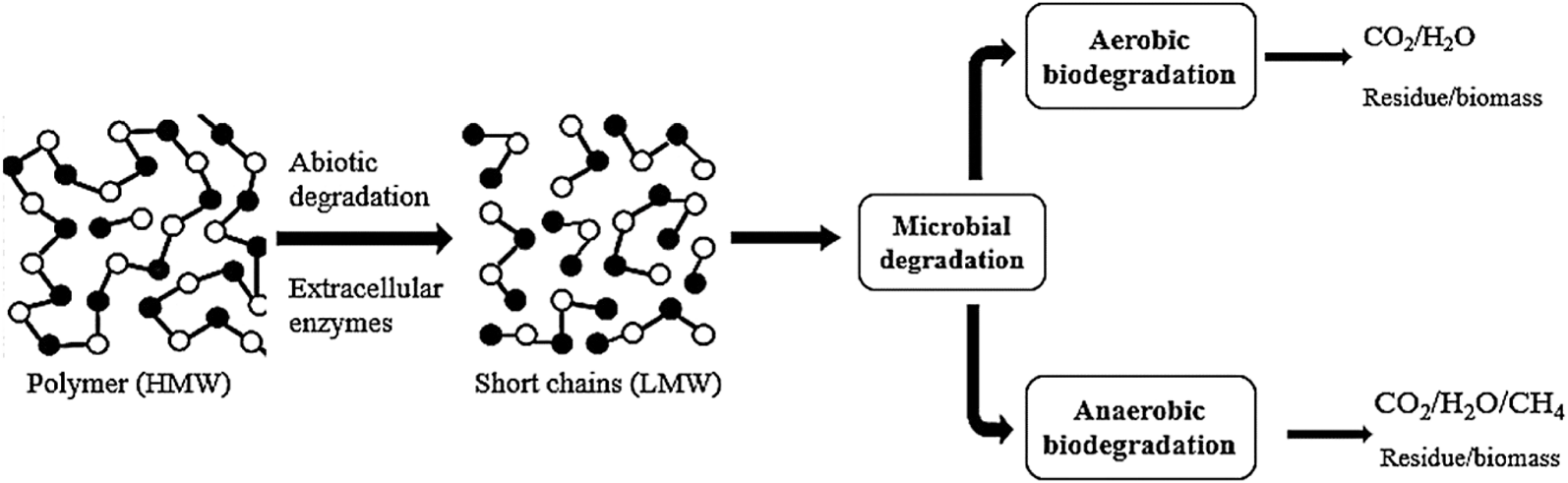
Schematic of polymer biodegradation into short-chain polymers. Published under the CC-BY License.^117^ Copyright 2022, the authors. Published by Nature.

Food packaging from recycled polymers and biodegradable polymers can mitigate pressing environmental issues.^110^ However, recycling plastic waste is complex due to its multistage processing, such as sorting, decontamination, and separation. Since food packaging requires a hygiene standard, recycling may not be suitable in this case.^111^ Rather, using biodegradable packaging materials for food packaging is a viable alternative since these are biodegradable or compostable and these materials do not need to incinerate, resulting in lower greenhouse gas emissions and reduced waste management cost.^110^ Most used biodegradable polymers are polylactic acid (PLA), poly-hydroxy alkanoate (PHAs), polybutylene succinate (PBS), poly(butylene adipate-*co*-terephthalate) (PBAT), polysaccharide, protein blends, *etc.*.^112^ Chemical functional groups play a major role in this. For example, ester, carbonyl, hydroxyl, and glycosidic linkages are more vulnerable to hydrolysis than the stable carbon–carbon backbone found in polyolefins.^112^ However, processing is also responsible for degradation. Lower crystallinity, lower molecular weight, higher hydrophilicity, and greater amorphous content generally improve water penetration and enzyme accessibility, allowing chain scission to proceed more efficiently.^12^ For this reason, aliphatic polyesters or carbohydrate-based polymers are widely studied for compostable packaging applications. Biodegradation mainly occurs through abiotic and microbial degradation pathways, where polymer chains are initially fragmented into low-molecular-weight compounds before undergoing further mineralization under aerobic or anaerobic conditions (Fig. 6). The biodegradability of polymers is strongly influenced by their chemical structure and physicochemical properties. Polymers containing hydrolysable functional groups such as ester, amide, carbonyl, hydroxyl, and glycosidic linkages are more susceptible to enzymatic and hydrolytic degradation than polymers with stable carbon–carbon backbones such as PE and PP.^113^ Lower crystallinity, lower molecular weight, higher amorphous regions, and increased hydrophilicity facilitate water penetration and microbial enzyme accessibility, accelerating polymer chain scission and biodegradation.^114^ In contrast, conventional petroleum-based polymers exhibit poor degradation due to their hydrophobic nature, high molecular stability, and dense crystalline structure.^115^ Despite the environmental advantages of biodegradable polymers, several limitations remain, including higher production cost, limited large-scale industrial scalability, inferior mechanical and barrier properties compared to conventional plastics, dependency on controlled composting conditions, insufficient composting infrastructure, concerns regarding food-contact safety, and the potential migration of additives or degradation by-products into food systems.^116^Table 8 shows the materials, production process, processing technologies, and application of biodegradable polymers.

**Table 8:** Materials, production process, processing technologies, and applications of biodegradable polymers

Polymer	Materials	Synthesis	Technologies	Applications	Ref.
PLA	Film and composites	Ring opening/condensation	Drying, extrusion, injection molding, blow molding, cast film extrusion, and thermoforming	Chicken meat preservation, PLA films, wrappings, laminates, and containers (bottles and cups)	118
PHAs	Film	Microbial biosynthesis and chemical synthesis	Solvent casting and melt blending	Packaging films, containers, shampoo bottles, shopping bags, and cups	119
PBS	Film	Condensation polymerization	Film extrusion, blown film extrusion, film casting, thermoforming, multilayer films, foams and blends, compression, and injection molding	Packaging films (fruits, vegetables, meat, seafood, dairy foods, bakery products, cereal, confectionary and beverages), trays, bottles, and other containers	120 and 121
PBAT	Film	Polycondensation	Solution casting, blown films, compression molding and casting, extrusion Blown, hot melt extrusion, blown film extrusion, and melt extrusion	Packaging films, garbage bags, pouches, packaging films, mulch films, and paper laminations	122
Polysaccharide blend	Film	—	Solvent casting, tape casting, extrusion, film blowing, electrospinning, forcespinning, 3D-printing, and reactive extrusion	Bags for fruits and vegetables	123
Cellulose- and derivative-based biopolymer	Coatings, films, composites, and nanocomposites	—	Solution casting, layer-by-layer assembly (LBL), extrusions, coatings, polymeric hydrogel, spraying (spray drying), electrospinning, micro- and nano-encapsulation, liposomes and nanoliposomes, nano micelles, nano emulsion, and adsorption	Packaging films (fruits, vegetables, meat, beef, and chicken preservation) and food containers	124
Proteins	Films/coatings	Open-air controlled radical polymerization	Solvent casting, 3-D printing, melt extrusion, and electrospinning	Food packaging and 3D printing	125

### 4.1 Bio-based biodegradable synthetic polymers

Bio-based biodegradable polymers derived from renewable or nonrenewable resources.^126^ Polycaprolactone (PCL), polyglycolic acid (PGA), and PBAT are biodegradable polymers from nonrenewable polymers, and PLA, PHA, PBS, and poly(butylene adipate-*co*-succinate) (PBAS) are from renewable source.^127^ Shiddique *et al.*^128^ converted cotton-based post-consumer waste into biodegradable paper for food packaging and other uses in replace of petroleum-plastic materials. Md. Abdus Shahid *et al.*^129^ developed a packaging material from jute-based nonwoven fabric. Jute caddis cellulose (JCC) from lignocellulose biomass waste can be used in preparing biodegradable films, and flexible, semi-transparent, biodegradable, and highly water-resistant eco-films have already been developed.^130^ Plant-based natural fibers can be used in food-packaging material preparation for their outstanding physico-chemical and mechanical properties. Table 9 shows different polymer categories with their raw sources and production methods.

**Table 9:** Direct raw sources of bio-based and conventional plastic polymers

Polymer	Category	Direct raw sources	Production method	Ref.
PLA	Biodegradable	Corn, sugarcane, cassava, and wheat (starch) → fermented to lactic acid	Bacterial fermentation → ring-opening polymerization	65
PHA	Biodegradable	Food waste, vegetable oils, wood sugars (*e.g.*, xylose), and methane (from oil/gas)	Microbial fermentation (*e.g.*, *Cupriavidus necator*)	131
PBS	Biodegradable	Petroleum or corn/sugarcane (succinic acid) + oil-derived 1,4-butanediol	Polycondensation	132
PBAT	Biodegradable	Petroleum (adipic acid, terephthalic acid) + corn (1,4-butanediol)	Polycondensation	133
Polysaccharide blends	Biodegradable	Corn, potato, cassava, and wheat (starch granules)	Blending with synthetic polymers (*e.g.*, PLA and PCL)	134
Bio-PE	Non-biodegradable	Sugarcane and corn (ethanol) → ethylene or petroleum (crude oil cracking)	Polymerization of ethylene	26
Bio-PP	Non-biodegradable	Sugarcane (ethanol) → propylene or petroleum (oil refining)	Polymerization of propylene	26
Bio-PET	Non-biodegradable	Sugarcane (ethylene glycol) + petroleum (terephthalic acid) or wood (HMF)	Polycondensation	35
Bio-PTT	Non-biodegradable	Corn (1,3-propanediol) + petroleum (terephthalic acid)	Polycondensation	35
Bio-PA (Nylon)	Non-biodegradable	Castor oil (PA 11), wood (PA 410), and petroleum (caprolactam for PA 6)	Polycondensation or ring-opening polymerization	37
Conventional PE/PP	Non-biodegradable	Petroleum (crude oil → naphtha)	Cracking → polymerization	25

#### 4.1.1 Polylactic acid (PLA)

PLA is polymerized from lactic acid (LA), also known as 2-hydroxypropanoic acid or α-hydroxypropionic acid, and serves as the primary monomer for PLA. LA is extracted through the fermentation of carbohydrate-rich feedstocks such as starch, rice, sugarcane, corn, food waste, and other polysaccharide materials.^135^ Tagudin and Ibrahim^135^ showed the synthesis of PLA from apple, pineapple, and potato residues. There are three main approaches used to synthesize PLA: direct polycondensation of lactic acid, azeotropic dehydrative polycondensation to facilitate water removal, and ring-opening polymerization of lactide, which is commonly employed in large-scale production.^136^

**Fig. 7:**
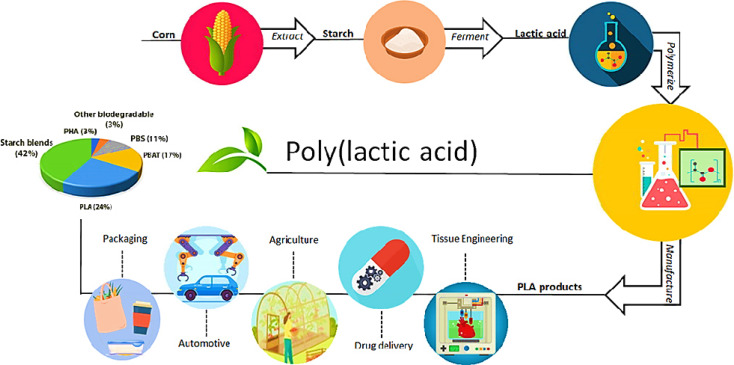
PLA production from corn and packaging development. Published under the CC-BY License.^141^ Copyright 2021, the authors. Published by MDPI.

Along with other applications, PLA is an emerging food-packaging polymer owing to its good mechanical performance, transparency, compostability, printability, heat sealability, hydrophilicity, *etc.*.^136^ It is commonly extruded or cast onto thin films and wraps, which are used for flexible food packaging. PLA is also thermoformed into rigid containers, including cups, trays, and clamshells for fresh produce and takeaway applications.^137^ L. Di Maio *et al.*^138^ developed biodegradable active PLA films. Tunable processing allows manufacturing of PLA packaging with desired mechanical properties needed for specific packaging materials. For example, PLA having more crystallinity has better chemical stability and water resistance.^136^ The most important feature that made PLA significant for packaging materials is their compostability and biodegradability. PLA degradation occurs in a multi-step mechanism that involves chemical and microbial processes.^139^ Colonized microorganisms include the incorporation of fungi and bacteria onto the polymer surface and breaking the polymers into small fragments. The hydrophilic groups of enzymes (-COOH, –OH, and –NH) attack the ester group by hydrolysis followed by oxidation reactions.^140^Fig. 7 presents the process of PLA production from starch.

#### 4.1.2 Poly-hydroxy alkanoate (PHA)

Polyhydroxy alkanoates (PHAs) are bioplastics commonly used in sustainable food packaging. They are sourced from a wide range of renewable carbon sources, including starches and sugars from crops such as corn, sugarcane, and cassava, as well as lignocellulosic biomass, agricultural residues, dairy by-products, food processing waste, fruit and vegetable waste, waste oils, glycerol, and organic-rich industrial wastewaters.^142^ The synthesis of PHAs is done by microorganisms through glycolysis of sugars and β-oxidation of fatty acids, depending on the carbon source. Production systems include halophilic bacteria and archaea grown on organic or carbon-rich waste streams, microalgae and macroalgae biomass as renewable feedstocks, and mixed microbial cultures (MMC) for cost-efficient waste valorization. It is not just a single type of polymer, rather a diverse class of biodegradable bioplastics, ranging from stiff and crystalline short-chain poly(3-hydroxybutyrate) (PHB), poly(3-hydroxybutyrate-*co*-3-hydroxyvalerate) (PHBV), flexible and rubbery medium-chain-length PHAs, to poly(3-hydroxyhexanoate) (PHH).^143^ By adjusting the monomer type and content, its properties can be tailored into different packaging needs, from rigid trays to compostable films.^144^

PHA films can achieve low oxygen permeability and reasonable moisture resistance under favorable conditions. With proper design (*e.g.*, copolymers, additives, or multilayers), their UV stability and barrier performance can be enhanced to suit food-packaging applications.^145^ Orimi H. *et al.*^146^ developed cellulose crystalline nanocomposite based on PHB obtained from *Cupriavidus necator* for developing the shelf life of food products. Cheng Z. *et al.*^147^ developed bamboo/PBAT bioplastic composite films, and PHBV coating applied to biodegradable substrates significantly reduced oxygen and water vapor permeability, by roughly 90%, demonstrating excellent barrier enhancement for multilayer or coated food-packaging structures. Organic waste streams represent viable and sustainable carbon and energy sources for the commercial production of polyhydroxyalkanoates (PHAs). However, the industrial cost of producing PHAs remains significantly higher than that of conventional plastics, typically ranging from $4 to $6 per kg compared to $1 to 2 per kg for petrochemical polymers. As a result, recent research has focused on utilizing low-cost waste feedstocks, strain engineering, and process intensification to reduce production costs.^148,149^

**Fig. 8:**
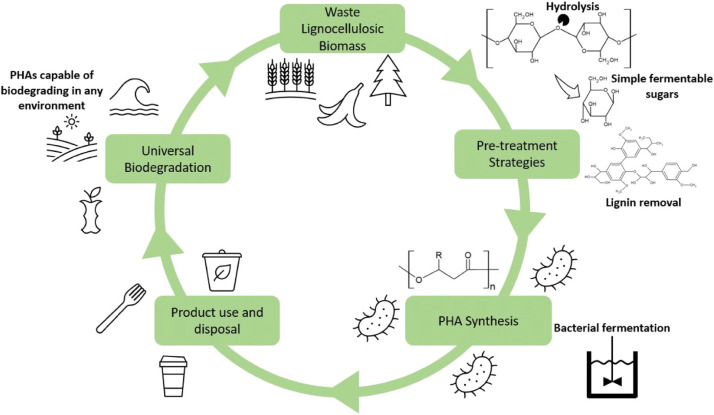
PHA production from lignocellulosic biomass and PLA films or materials developed through the bacterial fermentation process. Published under the CC-BY License.^152^ Copyright 2023, the authors. Published by the Royal Society of Chemistry.

PHAs are extremely quick in degradation both under aquatic and terrestrial environments, taking 9–12 days to lose significant amounts of mass.^150^ Under aerobic conditions, PHA polymers are degraded into water and carbon dioxide and, under anaerobic conditions, they degrade into methane and water.^151^Fig. 8 shows how the lifecycle of packaging plastics made from PHAs started from the synthesizing process of PHAs up to the biodegradation process.

#### 4.1.3 Polybutylene succinate (PBS)

**Fig. 9:**
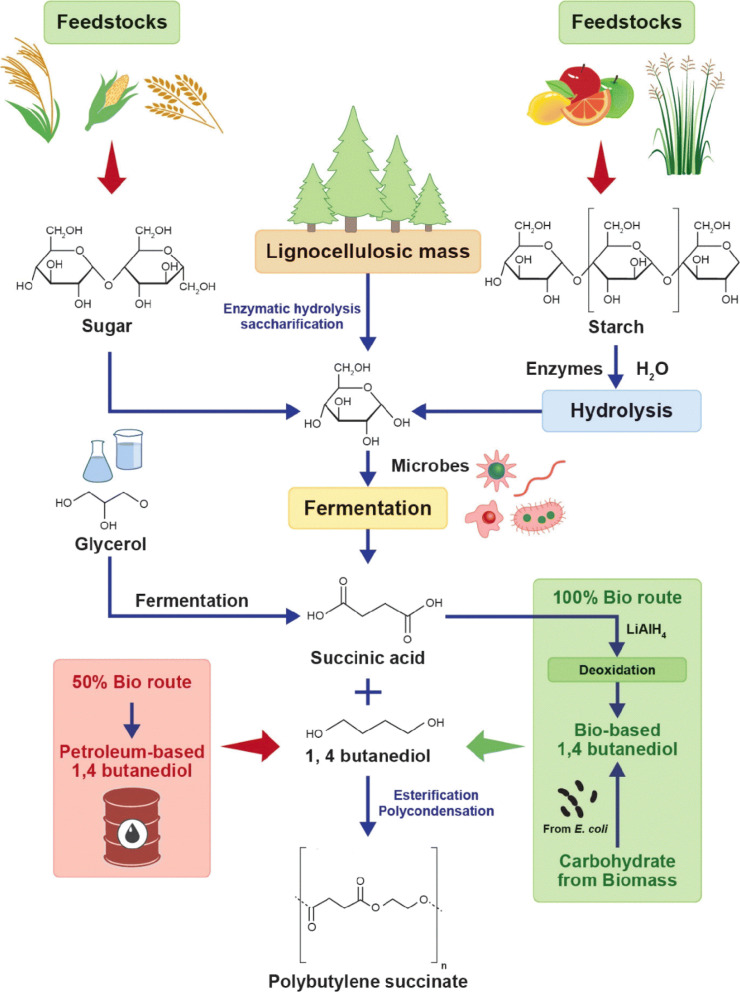
PBS production process from feedstock and lignocellulose mass. Published under the CC-BY License.^154^ Copyright 2025, the authors. Published by the Royal Society of Chemistry.

PBS and PBSA can be possibly produced from petrochemical sources and renewable sources such as sugarcane, cassava, corn, sugar, starch, and others lignocellulosic mass, as demonstrated in Fig. 9.^5^ PBS polymers are developed by the polycondensation of succinic acid (SA) and 1,4-butanediol (BDO) *via* a bacterial fermentation route.^153^ PBS polymer degrades through a two-stage process into water and carbon dioxide, followed by abiotic hydrolysis and microbial mineralization. In abiotic hydrolysis, long-chain polymer molecules break down into smaller water-soluble oligomers and monomers. In the second step involving microbial mineralization, microorganisms assimilate and mineralize these smaller fragments.^39^

PBS shows crystalline thermoplastic properties, and PBSA is more rigid and slightly ductile than PBSA. High-molecular-weight improves the mechanical properties; however, it slows down biodegradability. Blends of PLA and PBS improve the tensile properties and crystallinity. In several studies, the lowest crystallinity shows the highest degradation rates.^155^ Łopusiewicz, *et al.*^156^ developed bioactive PBS films modified with quercetin for food packaging, and Palmieri, F., *et al.*^157^ developed PBS composite films for food-packaging applications. PBS has other applications, such as drug release, tissue engineering, bone engineering, implants, film rolls, food containers, shopping bags, cutleries, sensors, film rolls, feeding pipes, biomedicine, and agricultural markets.^154^

#### 4.1.4 Poly butylene adipate-*co*-terephthalate (PBAT)

PBAT, a copolyester composed of aliphatic and aromatic groups, draws attention in the packaging industry due to its high flexibility among the biodegradable plastics. Due to its impressive elongation property of around 516%, this material functions like LDPE.^158^ Mold temperature, injection speed, and pressure will impact the mechanical properties of PBAT during production. Commercially available co-polyester PBAT are ECOFLEX® of Germany, ECOPOND® of China, Origo-Bi® of Italy, ECOWORD® and TUNHE of China. XINFU of China and so on.^159^

**Fig. 10:**
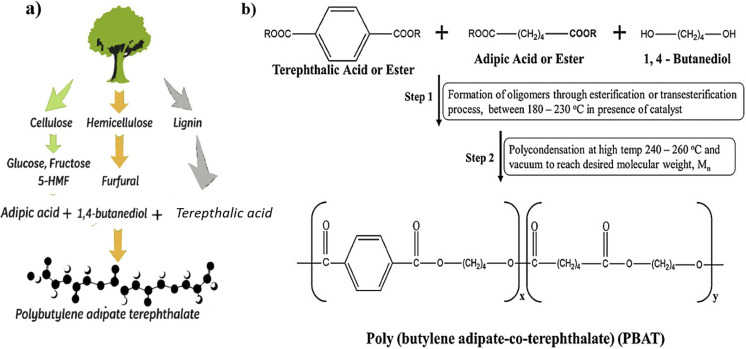
Schematic of (a) PBAT production from the adipic acid, 1,4-butanediol, and terephthalic acid blend developed from cellulose, hemicellulose, and lignin (published under the CC-BY License.^161^ Copyright 2024, the authors. Published by Chemistry Europe) and (b) PBAT production from terephthalic acid, adipic acid, and 1,4-butanediol by a polycondensation process at high temperatures (published under the CC-BY License.^162^ Copyright 2024, the authors. Published by Wiley).

The monomers of PBAT are derived from fossil-based sources as well as from bio-based sources, such as lignocellulosic forest residues, as illustrated in Fig. 10a.^160^ PBAT is then polymerized by condensation at high temperatures from 1,4-butanediol, adipic acid, and terephthalic acid, as shown in Fig. 10b. The degradation of PBAT occurs through two main pathways: abiotic processes (such as hydrolysis and photo-oxidation) and biotic processes (microbial biodegradation), whereas in marine environments, these mechanisms often act simultaneously, leading to gradual polymer breakdown.^133^ Microorganisms degrade PBAT into carbon dioxide and water within 7 weeks.^159^

A polymer blend with PBAT reduces the overall material cost, ensures the total biodegradability and sometimes increases the rate of biodegradability. PBAT composites are fabricated by *in situ* polymerization, melt mixing, and solvent casting methods.^159^ Venkatesan *et al.*^163^ developed PBAT composites with N,P-doped carbons for food packaging, Olonisakin *et al.*^164^ developed a PBAT-Lignin-Tannic acid composite film for the packaging of dry food products, Liu *et al.*^165^ developed antibacterial starch-based PLA/PBAT active packaging films for enhanced beef preservation, and PBAT is also used in mulch film, courier bags, and cutlery.^159^

### 4.2 Nature-derived biopolymers

Carbohydrates and proteins represent two of the most extensively investigated groups of naturally sourced biopolymers for packaging applications. Their wide availability, relatively low cost, and inherent biodegradability make them strong candidates, replacing conventional synthetic plastics in the development of sustainable packaging systems. Polysaccharide is a relatively large subgroup of carbohydrates used for packaging materials that include cellulose, starch, and chitin used for making biodegradable and sustainable packaging. On the other hand, proteins are sustainable macromolecules derived from animal sources such as keratin, casein, and gelatin.^166^

**Fig. 11:**
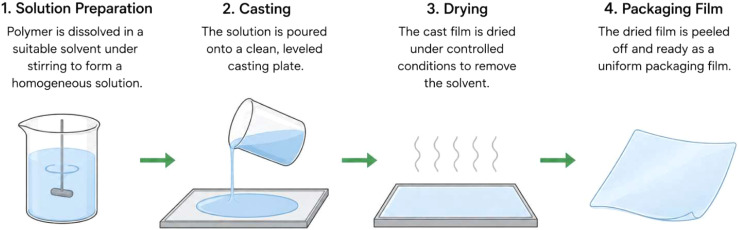
Solvent-casting process for biodegradable packaging film production: (1) polymer solution preparation, (2) film casting, (3) solvent evaporation and film formation, and (4) recovery of the finished biodegradable packaging film.

Biopolymers are mostly processed by solvent-casting methods to develop films for packaging materials, as shown in Fig. 11. Rincón *et al.*^167^ developed polysaccharide films derived from bay tree pruning waste for active food packaging, and Kafashan *et al.*^168^ developed a polysaccharide ternary nanocomposite based on basil seed gum/graphene oxide/anthocyanin for intelligent food packaging. Qamar S. *et al.*^169^ designed functional bioplastic films from cocoa shell cellulose and waxes. Abookleesh *et al.*^125^ designed protein-based bioplastic nanocomposite films for food packaging. Edible food-packaging films can be possible in food packaging from chitosan and protein-based biopolymers such as gelatin. Much research has been designed on biopolymeric coating focused on polysaccharide and protein, and those coatings are also edible.^147^ Keratin is an abundant protein found in animal hair and bird feathers, which can be used to make biodegradable food-packaging films;^170^ however, this protein-based biopolymer needs some other polymers or crosslinking agents to enhance the strength or impart functional properties for packaging applications,^171^ and blending improves water resistance, processing properties and antimicrobial properties. For example, nanocellulose blended with starch enhances the starch film properties and the mechanical properties.^66^

Packaging materials derived from cellulose undergo enzymatic cleavage by celluloses, producing glucose as a final degradation product. Additionally, packaging bags made of regenerated cellulose fibers also show rapid degradation in both soil and the marine environment.^172^ Similarly, polysaccharides such as starch are rapidly hydrolyzed by amylolytic enzymes, leading to complete mineralization under composting and soil conditions.^173^ Malekzadeh *et al.*^174^ studied starch/nanocellulose films under 30-days soil burial and observed 35–67% degradation, showing that starch-containing films are indeed susceptible to enzymatic hydrolysis in soils. Chitin-based materials degrade through the action of chitinases and deacetylases, yielding *N*-acetylglucosamine monomers. Protein-derived materials, including keratin and gelatin, are broken down through proteolytic enzymatic activity, producing amino acids that are easily assimilated by microorganisms.^175^

### 4.3 Bio-based additives

Additives derived from bio-based sources are deemed non-toxic, sometimes edible,^176^ and safe for use in the food-packaging application. Extensive research has been conducted to find such safe and environmentally friendly additives to replace non-degradable, toxic chemicals while uncompromising their functionality and properties. These additives range from plasticizers, antioxidants, stabilizers, to anti-microbial agents. Naturally derived polyols such as glycerol, sorbitol, mannitol, and xylitol have shown potential as plasticizers. Additionally, vegetable- and lipid-derived biomolecules such as soybean oil, modified linseed oil, cottonseed oil, castor oil, and sunflower oil can be used as plasticizers for biodegradable polymers such as starch, PLA, PHA, and PHBV.^177^ Wadhi and Weliam^178^ used sunflower oil (SO) to plasticize solvent-cast PLA films for food packaging and found almost 200% elongation at break when 20% sunflower oil was blended with 80% PLA while without SO, neat PLA showed 43% elongation at break. The modulus of neat PLA also found to decrease with the increase in SO concentrations. Maleinized linseed oil (MLO) was used as a plasticizer to improve the ductility of PLA and thermoplastic starch (TPS) melt-extruded films. MLO reduces intermolecular interaction of PLA/TPS by inserting them within a polymer matrix, resulting in enhanced free volume. With the increase in MLO yield, the flexural strength and damping factor was found to be decreased indicating effective plasticization.^179^

**Fig. 12:**
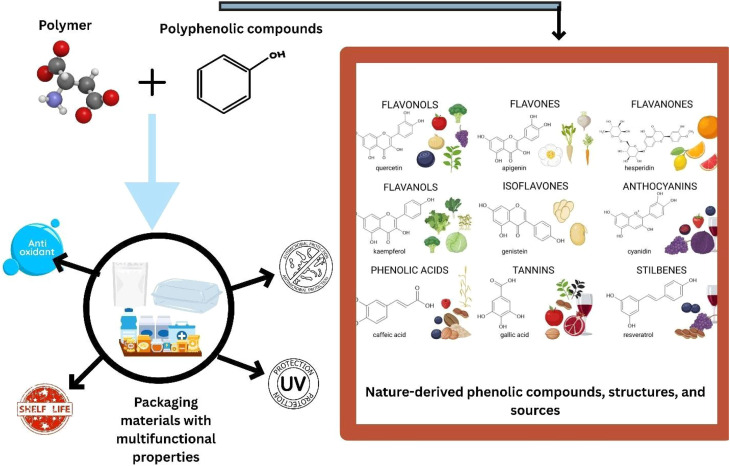
Effects of nature-derived polyphenols (shown in the square box) as multifunctional additives for food packaging. Published under the CC-BY License.^185^ Copyright 2024, the authors. Published by MDPI.

Using potential carcinogenic antioxidants, such as *tert*-butylhydroquinone (TBHQ), propyl gallate (PG), and butylated hydroxytoluene (BHT), synthetic phenolic compounds can be replaced by naturally derived essential oils, natural polyphenols, and phospholipids.^117^ Essential oils such as cinnamon oil, ginger oil, rosemary oil, and lavender oil have shown excellent antioxidant properties of films when blended with polysaccharide, protein, and starch.^180^ These molecules work as scavengers of free radicals or metal chelating agents and therefore inhibit lipid oxidation. Some of those also work as antimicrobial, UV-resistant agents, offering multiple actions, as shown in Fig. 12. For example, nature-derived polyphenolic tannic acid improved the barrier performance by lowering the oxygen permeability of chitosan films from 1.63 × 10^−4^ to 1.53 × 10^−4^ and that of gelatin films from 1.51 × 10^−4^ to 1.36 × 10^−4^ cc m per day per atm and significantly enhanced antimicrobial activity, *e.g.*, *E. coli* inhibition increased from 7 mm to 15 mm in chitosan films and from 0 mm to 8 mm in gelatin films.^181^ Nature-derived polyphenols can be blended, sprayed, or coated onto the packaging materials to inhibit oxidation and enhance the shelf life, antioxidant activity, antimicrobial activity, *etc.* Chitosan itself works as an antimicrobial agent for food packaging. Bie *et al.*^182^ demonstrated that incorporating chitosan into poly(lactic acid)/starch blends produced films with a sustained release profile. Chitosan diffused in two stages: an initial rapid release followed by a slower, prolonged phase. This dual mechanism enabled both immediate microbial inhibition and long-lasting antimicrobial protection, making the films suitable for high-moisture foods such as fresh meat. Chitosan-infused biodegradable PVA films effectively inhibited the growth of *E. coli* and *Listeria monocytogenes*, two common foodborne pathogens, extend the shelf life of meat.^183^ Besides, nature-derived polyphenolic compounds such as flavonoids (such as quercetin and anthocyanins), phenolic acids (gallic acid, caffeic acid, and ferulic acid), tannins, stilbenes (resveratrol), lignans, and coumarins are excellent antimicrobial agents for food packaging.^184^

## 5 Strategic approach

### 5.1 Policy intervention

The UNEP Plastics Initiative has four goals: reducing the size of the problem, design for circularity, ensuring circularity in practice, and dealing with legacy. In 2021, the EU proposed the action plan “Towards zero pollution for air, water and soil” and will reduce 30% microplastic release into the environment by 2030. On 25 September 2023, the EU adopted a regulation on the restriction of microplastics intentionally added to products.^186^ 45 countries, 500 private sector actors and 50 financial institutions have targeted to include circularity or plastic pollution prevention and reduction policies recommended by UNEP by 2030.^187^ The objective of regulation (EU) 2024/1991 is to implement the targets set by the European green deal and EU biodiversity strategy for 2030. The EU targeted to restore at least 20% of the degraded ecosystem by 2030 and 60% by 2050. EU member states must submit their green plan by June 2025.^188^

### 5.2 Consumer awareness

Plastic pollution is considered a challenge in the world. Not only government rules and regulations but also consumer awareness is necessary to control plastic pollution. A research project on consumer awareness was published, where a total of 103 interviews were conducted with 124 participants from December 2018 and February 2019. Consumer awareness varies among individuals, and most of the consumers recognize the issues associated with plastic waste and through interviews, five distinct types of awareness were identified. Identified awareness is, respectively, awareness of environmental pollution, awareness of the intensive use of plastic, awareness of consumers' influence, awareness of consumers' powerlessness, and awareness of the necessity of plastic. A statement from a consumer*,* “I would pay more for environmentally friendly packaging alternatives. I have three grandchildren and they should still be able to live in this world. I would also pay more for environmentally friendly packaging alternatives if it were good for the environment and our grandchildren! they will suffocate in the waste”.^189^ Growing awareness of consumers will drive the packaging industry to adopt sustainable pathways, contributing to minimizing pollution and health issues.

## 6 Conclusions

Modern food-packaging industry is trying to reduce the production cost of food-packaging materials, and thus, plastic-based synthetic food-packaging materials, such as PE, PP, PET, PTT, and PA, have caught attention. Although inexpensive and easy to process, their environmental and health costs are substantial. This paper highlighted the environmental and health hazard caused by the synthetic non-biodegradable packaging and illustrated possible solutions based on the existing literature.

Different forms of environmental pollution of synthetic food packaging stem from leaving carbon footprint landfills, non-degradability, and microplastic release into aquatic environments. These materials contribute to greenhouse gas emissions across their life cycle, shed microplastics that destabilize aquatic ecosystems, and persist in landfills where their poor degradability undermines soil health and accelerates waste accumulation. These adverse effects are caused not only by the synthetic packaging materials but also by the use of synthetic additives during processing. More than 10 000 hazardous chemicals are detected in the marine environment, human food chain, and others, where a significant portion is relevant to the packaging industry. Poisonous heavy elements such cadmium, mercury, and arsenic and toxic migrants such as bisphenol A, phthalates, styrene, caprolactam, vinyl chloride, dioxin, paraben, and perfluoroalkyl substances were found in synthetic food-packaging materials. Microplastic, heavy metal, and toxic migrants are responsible for different health issues, such as cancers, like lung cancer, reproduction system damage, brain damage, skin damage, allergic reactions, and DNA damage.

The replacement of synthetic polymers with biodegradable alternatives for food-packaging materials is a promising approach to mitigate pressing environmental problems associated with plastic packaging. Biodegradable materials such as PLA, PHAs, PBS, and PBAT are bio-based polyesters and reported to degrade easily in soil and industrial composting systems. The application of bio-based biodegradable prime raw materials in manufacturing food-packaging materials and bio-based molecules as additives would reduce energy consumption, disposal problems, carbon emissions and microplastic release. Abundant biopolymers, especially agricultural waste, including cellulosic and protein wastes, are highly effective in this case; however, they suffer from scalability and strength limitations. Advancing this transition will require deeper exploration of underused biomass streams, careful assessment of production feasibility and cost, and a coordinated push from policy and public awareness. Together, these efforts can accelerate the shift toward sustainable food-packaging systems that meaningfully reduce environmental burden and health risk.

## Author contributions

Md. Amir Khasru: conceptualization, methodology, data analysis, writing – original draft, visualization; Tarikul Islam: conceptualization, methodology, writing – original draft, visualization, supervision, writing – review and editing; Sayam: writing – original draft, visualization, writing – review and editing; Md Shakirul Islam: investigation, writing – original draft, visualization, writing – review and editing.

## Conflicts of interest

The authors declared that they have no conflicts of interest.

## Data Availability

No new data were generated in this review. Therefore, data sharing is not applicable.
